# **Characterization of Polyvinyl Alcohol–Collagen–Hydroxyapatite Composite Membrane from** ***Lates calcarifer*** **Scales for Guided Tissue and Bone Regeneration**

**DOI:** 10.1055/s-0042-1759488

**Published:** 2023-01-16

**Authors:** Yessy Ariesanti, Putri Graesya Melani Octavianus, Annisa Tri Handayani, Basril Abbas

**Affiliations:** 1 Department of Oral and Maxillofacial Surgery, Faculty of Dentistry, Universitas Trisakti, Jakarta, Indonesia; 2 Undergraduate Program, Faculty of Dentistry, Universitas Trisakti, Jakarta, Indonesia; 3 National Research and Innovation Agency (BRIN), Jakarta, Indonesia

**Keywords:** guided tissue regeneration, guided bone regeneration, PVA–collagen–HA composite membrane, tensile strength, chemical structure, porosity, degradability

## Abstract

**Objective** To determine the chemical structure, tensile strength, porosity, and degradability of polyvinyl alcohol (PVA)–collagen–hydroxyapatite (HA) composite membranes for guided tissue and bone regeneration.

**Materials and Methods** The PVA–collagen–HA composite membrane was divided into three groups: the group without irradiation, the group with 15 kGy irradiation, and 25 kGy irradiation. Each group was tested for chemical structure with Fourier-transform infrared (FT-IR) at a wavelength of 400 to 4,000 cm ^−1^ . Tensile strength test was tested in dry and wet conditions with the standard method of American Standard Testing Mechanical (ASTM) D638, and porosity using scanning electron microscope and analyzed using ImageJ software. Degradability test immersed in a solution of phosphate-buffered saline. Data were analyzed using analysis of variance (ANOVA) and Tukey's test.

**Results** FT-IR test before and after storage for 30 days on three media showed a stable chemical structure with the same functional groups. ANOVA analysis showed a significant difference ( *p* < 0.05) in the dry condition ( *p* = 0.006), Tukey's test showed a significant difference in the 15 kGy and 25 kGy irradiated groups ( *p* = 0.005), but the groups without irradiation had no significant difference with the 15 kGy ( *p* = 0.285) and 25 kGy ( *p* = 0.079) irradiation groups. In wet conditions, there was no significant difference ( *p* > 0.05) in each group ( *p* = 373). The size of the porosity in the group without irradiation, 15 kGy irradiation, and 25 kGy irradiation showed a size of 4.65, 6.51, and 8.08 m, respectively. The degradability test showed a decrease in weight in each group, with the total weight of the membrane being completely degraded from the most degraded to the least: the groups without irradiation, 15 kGy irradiation, and 25 kGy irradiation. The ANOVA test on the degradability test shows significant ( *p* < 0.05) in the PVA–collagen–HA composite membrane group over time intervals ( *p* = 0.000). Tukey's post hoc test showed a significant difference ( *p* < 0.05) after 1 week between the groups without irradiation with 15 kGy ( *p* = 0.023).

**Conclusion** PVA–collagen–HA composite membrane has a stable chemical structure, optimal tensile strength, porosity, and ideal degradability as guided bone regeneration and guided tissue regeneration.

## Introduction

Tooth extraction is a surgical procedure in dentistry to remove a tooth from its socket. 1 After tooth extraction, the socket often experiences wounds that begin with bleeding, then the socket wall will stimulate the wound healing process and regenerate soft and hard tissues. However, this healing process cannot completely restore normal anatomic structures because of physiological changes due to alveolar bone resorption. 2 3

Alveolar bone resorption can affect the determination of indications and prognosis in the installation of dental implants. 4 A poor prognosis in installing dental implants can be avoided by preserving the socket with a guided tissue regeneration (GTR)/guided bone regeneration (GBR) approach that functions as a membrane barrier. The barrier membrane can prevent epithelial cells and connective tissue from entering the defect area so that it can support optimal soft and hard tissues regeneration. 5 6

Barrier membrane must have several requirements, including the integrity of the membrane surface structure, chemical properties, mechanical properties, and physical properties to support vascularization, wound stabilization, maintain blood clots, and prevent connective tissue and epithelial cells from entering the defect area. This will support optimal soft and hard tissues regeneration. 7 Chemical properties of a barrier membrane must have a stable chemical structure in the bonds between the polymers contained. GTR/GBR membranes must have good mechanical properties, that is, tensile strength capable of providing elastic, flexible, and strong enough properties to maintain structural integrity and withstand the tensile strength of tissue and bone. 8 The ideal physical property has a porosity that can prevent soft tissue growth into the socket. Based on the size, porosity is divided into two, namely, microporous (≤10 µm) and macroporous (≥100 µm). 9 Microporosity can increase macromolecular adhesion and support liquid penetration. 10 In a study of a membrane with a microporous size, it was more effective in regenerating bone than a membrane with a large pore. 11 A membrane barrier must have an ideal degradability time. In soft tissue, it takes 4 to 6 weeks for soft tissue regeneration, and in hard tissue, it takes 12 to 24 weeks for bone regeneration. 12 13

Composite membrane biomaterials consist of natural and synthetic polymers designed to meet physical, chemical, and mechanical properties that can support soft and hard tissues regeneration. 14 One of the biomaterials that have been carried out to support the regeneration of soft and hard tissues is the polyvinyl alcohol (PVA)–collagen–hydroxyapatite (HA) composite membrane made from white snapper ( *Lates calcarifer* ) scales. Based on the results of previous studies, it was shown that the PVA–collagen–HA composite membrane had the potential for GTR 15 and GBR. 16 As an ideal membrane requirement, the PVA–collagen–HA composite membrane must have a stable chemical structure, optimal tensile strength, porosity, and ideal degradability. This research will investigate the ideal chemical structure, tensile strength, porosity, and degradability of the PVA–collagen–HA composite membrane.

## Materials and Methods

### PVA–Collagen–HA Composite Membrane Manufacturing

**Fig. 1:**
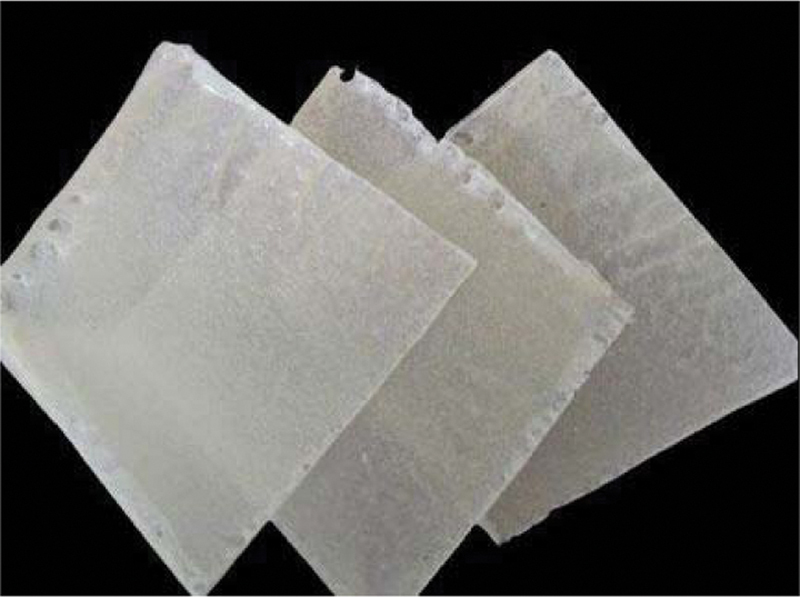
Polyvinyl alcohol–collagen–hydroxyapatite composite membrane.

In this study, the PVA–collagen–HA composite membrane ( Fig. 1 ) was made from a synthetic polymer, namely, PVA (Polyvinyl alcohol 72000, Merck-Schuchardt OHG, Hohenbrunn, Germany), and natural polymers, namely, collagen and HA extract from white snapper scales ( *L. calcarifer* ) which is processed by chemical hydrolysis at the National Research and Innovation Agency (BRIN), South Jakarta, Indonesia (No: IDP00007025). After that, irradiation was carried out at doses of 15 kGy and 25 kGy based on ISO 11137 using a gamma cell irradiator. In this study, they were divided into three groups: composite membranes (PVA–collagen–HA) without irradiation (control), 15 kGy irradiation, and 25 kGy irradiation.

### Functional Group Analysis with Fourier Transform Infrared

The chemical structure was identified using Fourier transform infrared (FT-IR) spectroscopy analysis by measuring the absorption of infrared radiation at various wavelengths to determine the functional groups of the compounds contained in the PVA–collagen–HA composite membrane. The test was carried out in two phases, phase 1 at the beginning of the study and phase 2 after storage for 30 days on three media. A PVA–collagen–HA composite membrane with a size of 5 × 5 mm was placed on a disc and inserted into an FT-IR Spectrophotometer (IR-Prestige-21, Shimadzu, Tokyo, Japan) with an infrared wavelength of 400 to 4,000 cm ^−1^ , resolution 2.0, and measurement mode percent (%) transmittance.

### Tensile Strength Test

Tensile strength was measured using a Universal Testing Machine (UTM) (Xforce P(Z005), Zwick Roell, Germany) based on the American Standard Testing Mechanical (ASTM) D638 standard method. The sample was printed 7 × 7 cm with a Dumbbell Die Cutter (SDL-100, Dumbbell Co, Saitama-Ken, Japan) according to the ASTM D1822L standard method. The research sample in the tensile strength test was divided into two conditions, namely, the PVA–collagen–HA composite membrane in dry conditions and wet conditions soaked with saline sterile NaCl solution (0.9% sodium chloride, PT Widatra Bhakti, Pasuruan, Indonesia). The PVA–collagen–HA composite membrane was placed on a UTM device with a tensile load and a speed of 0.1 N and 50 mm/min in dry conditions and 20 N in wet conditions at a speed of 100 mm/min.

### Porosity Test

Each group was cut with a size of 5 × 5 mm. The surface of the PVA–collagen–HA composite membrane was coated with 10-nm-thick gold using a sputter coater (Quorum Q150R ES, Quorum, East Sussex, UK) and observed using a scanning electron microscope (SEM) (SEM EVO MA 10, ZEISS, Jena, Germany) at an accelerating voltage of 16 kV. The photo taken is the membrane surface with ×1,000 and ×5,000 magnification. The interpretation results of SEM were analyzed using ImageJ software.

### Degradability Test

Each group was cut with a size of 1 × 1 cm, placed in plastic medicine bottles containing 10 mL of phosphate-buffered saline (PBS) (Merck KGaA, Darmstadt, Germany), and placed in an incubator (Heraeus, Hanau, Germany) at 37°C, observed at intervals of 7, 14, 21, and 28 days. Each group was taken according to the time interval and dried at room temperature 25°C for 4 days until no weight variation was detected. Each group was weighed using an analytical balance (GR200, A&D Company Limited, Tokyo, Japan), and the weight was recorded. The weight ratio of each membrane will be calculated using weight ratio formula to obtain the degradability value. The number of each group in the sample of this study was repeated six times ( *n* = 6).

Weight ratio formula 17 :

Weight ratio (%) = *W* 1/ *W* 0 × 100

*W* 1 = current weight after the relegation interval.

*W* 0 = initial weight.

Each group of PVA–collagen–HA composite membranes will calculate the total weight of the degraded membrane using the linear equation 18 :

*ax* + *by* = *c*

### Statistical Data Analysis

Normality test was done by using Kolmogorov–Smirnov test. Then it was analyzed using statistical tests (IBM SPSS Statistics, 28.0.1.1) one-way analysis of variance (ANOVA) if the results were significant ( *p* < 0.05), then followed by the Tukey's post hoc test.

## Result

### FT-IR Test Results

**Fig. 2:**
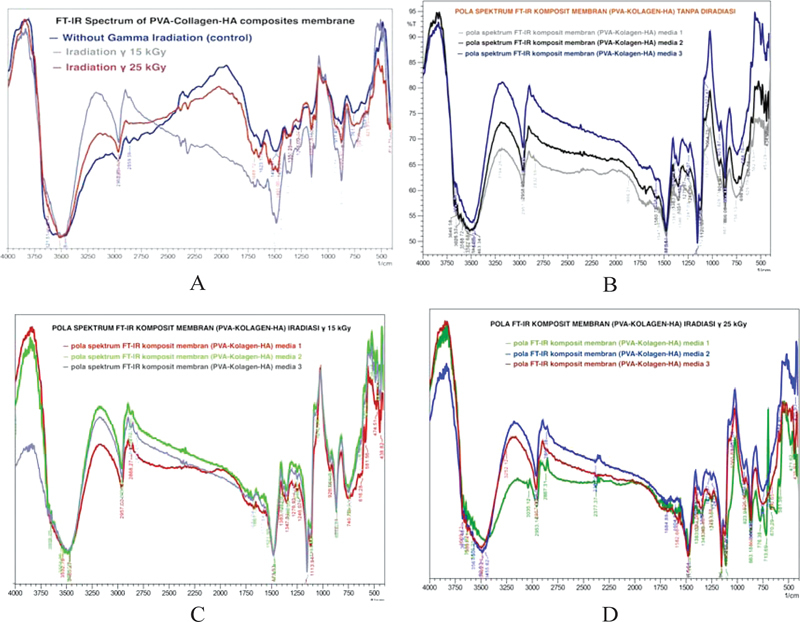
Infrared spectrum graph of the results FT-IR test PVA–collagen–HA composite membrane. (A) Phase 1 in the group without irradiation (control), irradiation 15 kGy, and irradiation 25 kGy. (B) Phase 2 FT-IR test on medium 1, medium 2, and medium 3 without irradiation. (C) Phase 2 FT-IR test on medium 1, medium 2, and medium 3 irradiation 15 kGy. (D) Phase 2 FT-IR test on medium 1, medium 2, and medium 3 irradiation 25 kGy. FT-IR, Fourier transform infrared; HA, hydroxyapatite; PVA, polyvinyl alcohol.

The graph of the infrared spectrum of the results of the FT-IR phase 1 test on the PVA–collagen–HA composite membrane group without irradiation (control), 15 kGy irradiation, and 25 kGy irradiation ( Fig. 2A ) shows the presence of PVA, collagen, and HA functional groups indicated by characteristic peaks. It appeared after being analyzed by standard vibrational regions based on spectrophotometric identification ( Table 1 ). The FT-IR phase 1 test results showed that at the peak of the PVA characteristics, OH strain was found with solid and comprehensive absorption and asymmetric CH strain with moderate absorption. At the characteristic peak of collagen, we found NH stretching vibrations (amide A) with weak absorption, C=O stretching or amide I vibrations with weak absorption, and NH bending vibrations combined with CN stretching vibrations of the peptide bond (amide II), which gave weak absorption. In addition, at the peak of the characteristic HA, we found an appeal to the moderate uptake P=O aliphatic group.

**Table 1:** Identification of characteristic peaks in FT-IR test of PVA–collagen–HA composite membrane phase 1

Functional groups	Characteristic peaks in the vibrational region (cm ^−1^ ) and intensity			Vibration area standard (cm ^−1^ )
	Phase 1				
	Without radiation	15 kGy	25 kGy	
Primary NH (amide A)	3,621.51; w	∼ 3,550; w	3,623.44; w	3,700–3,300 (w)
OH	∼ 3,424; br, s	3,499.99; br, s	3,512.52; br, s	3,600–3,500 (m/sh); 3,450–3,200 (s/br)
CHsp 3	2,960.86; m	2,958.93; m	2,961.82; m	2,950–2,800 (m)
Amide I	1,623.17; w	∼ 1,600; w	∼ 1,630; w	1,680–1,600 (w)
P=O aliphatic	1,150.59; m	1,151.55; m	1,152.52; m	∼ 1,150 (m)
Amide II	1,492.97; w	1,481.39; w	∼ 1,480; w	1,575–1,480 (w)

Abbreviations: br, broad; FT-IR, Fourier transform infrared; HA, hydroxyapatite; m, medium; PVA, polyvinyl alcohol; s, strong; sh, sharp; w, weak.

Infrared spectrum graph of the results of the FT-IR phase 2 test on the PVA–collagen–HA composite membrane without radiation (control) stored on three media, namely, medium 1 at room temperature without being exposed to light, medium 2 at room temperature exposed to light, and medium 3 in the refrigerator at 4°C ( Fig. 2B ) showed the presence of PVA, collagen, and HA functional groups based on the characteristic peaks that appeared. The FT-IR test identification of the PVA–collagen–HA composite membrane phase 2 showed the same functional groups as the identification of the functional groups that appeared on the spectrum graph with the functional groups that appeared in the FT-IR test phase 1. This proves that the chemical structure of PVA, collagen, and HA in the PVA–collagen–HA composite membrane group without irradiation was stable after being stored on the three media for 30 days ( Table 2 ).

**Table 2:** Identification of characteristic peaks in the FT-IR test of PVA–collagen–HA composite membranes without irradiation

Functional groups	Characteristic peaks in the vibrational region (cm ^−1^ ) and intensity				Vibration area standard (cm ^−1^ )
	Phase 1	Phase 2			
		Medium 1	Medium 2	Medium 3	
Primary NH (amide A)	3,621.51; w	3,655.26; w	3,646.58; w	3,663.94; w	3,700–3,300 (w)
OH	∼ 3,424; br, s	3,512.52; br, s	∼ 3,525; br, s	∼ 3,480; br, s	3,600–3,500 (m/sh); 3,450–3,200 (s/br)
CHsp 3	2,960.86; m	2,957.97; m	2,958.93; m	2,957.00; m	2,950–2,800 (m)
Amide I	1,623.17; w	∼ 1,680; w	∼ 1,690; w	∼ 1,680; w	1,680–1,600 (w)
P=O aliphatic	1,150.59; m	1,150.59; m	1,150.59; m	1,150.59; m	∼ 1,150 (m)
Amide II	1,492.97; w	1,547.94; w	∼ 1,570; w	1,574; w	1,575–1,480 (w)

Abbreviations: br, broad; FT-IR, Fourier transform infrared; HA, hydroxyapatite; m, medium; PVA, polyvinyl alcohol; s, strong; sh, sharp; w, weak.

Infrared spectrum graph of the results of the FT-IR phase 2 test on a 15 kGy irradiated PVA–collagen–HA composite membrane on three media, namely, medium 1 at room temperature without being exposed to light, medium 2 at room temperature exposed to light, and medium 3 in a refrigerator with a temperature of 4°C ( Fig. 2C ) indicated the presence of PVA, collagen, and HA content based on the characteristic peaks that appeared. The results of identifying the FT-IR test phase 2 on the 15 kGy irradiated PVA–collagen–HA composite membrane showed the same functional groups as the identification of the functional groups that appeared on the spectrum graph with the functional groups that appeared in the FT-IR test phase 1. This proves that the chemical structure of PVA, collagen, and HA on the 15 kGy irradiated PVA–collagen–HA composite membrane was stable after being stored on the three media for 30 days ( Table 3 ).

**Table 3:** Identification of characteristic peaks in the FT-IR test of PVA–collagen–HA composite membrane irradiated 15 kGy

Functional groups	Characteristic peaks in the vibrational region (cm ^−1^ ) and intensity				Vibration area standard (cm ^−–1^ )
	Phase 1	Phase 2			
		Medium 1	Medium 2	Medium 3	
Primary NH (amide A)	∼ 3,550; w	∼ 3,650; w	3,656.23; w	3,653.37; w	3,700–3,300 (w)
OH	3,499.99; br, s	∼ 3,499; br, s	∼ 3,500; br, s	3,474.81; br, s	3,600–3,500 (m/sh); 3,450–3,200 (s/br)
CHsp 3	2,958.93; m	2,957.00; m	2,956.04; m	2,956.04; m	2,950–2,800 (m)
Amide I	∼ 1,600; w	∼ 1,680; w	1,661.75; w	1,681.04; w	1,680–1,600 (w)
P=O aliphatic	1,151.55; m	1,150.59; m	1,150.59; m	1,150.59; m	∼ 1,150 (m)
Amide II	1,481.39; w	∼ 1,480; w	1,521.9; w	∼ 1,570; w	1,575–1,480 (w)

Abbreviations: br, broad; FT-IR, Fourier transform infrared; HA, hydroxyapatite; m, medium; PVA, polyvinyl alcohol; s, strong; sh, sharp; w, weak.

Infrared spectrum graph of the results of the FT-IR phase 2 test on a 25 kGy irradiated PVA–collagen–HA composite membrane on three media, namely, medium 1 at room temperature without being exposed to light, medium 2 at room temperature exposed to light, and medium 3 in a refrigerator with a temperature of 4°C ( Fig. 2D ) indicated the presence of PVA, collagen, and HA content based on the characteristic peaks that appeared. The identification of the FT-IR test phase 2 on the 25 kGy irradiated PVA–collagen–HA composite membrane showed the same functional groups as the identification of functional groups that appeared on the spectrum graph with the functional groups that appeared in the FT-IR test phase 1. This proves that the chemical structure of PVA, collagen, and HA on the 25 kGy irradiated PVA–collagen–HA composite membrane was stable after being stored on the three media for 30 days ( Table 4 ).

**Table 4:** Identification of characteristic peaks in the FT-IR test of PVA–collagen–HA composite membrane irradiated 25 kGy

Functional groups	Characteristic peaks in the vibrational region (cm ^−1^ ) and intensity				Vibration area standard (cm ^−1^ )
	Phase 1	Phase 2			
		Medium 1	Medium 2	Medium 3	
Primary NH (amide A)	3,623.44; w	3,635.97; w	3,676.48; w	3,651.41; w	3,700–3,300 (w)
OH	3,512.52; br, s	∼ 3,450; br, s	3,482.63; br, s	3,492.27; br, s	3,600–3,500 (m/sh); 3,450–3,200 (s/br)
CHsp 3	2,961.82; m	2,953.14; m	2,956.04; m	2,957.00; m	2,950–2,800 (m)
Amide I	∼1,630; w	∼ 1,680; w	1,684.89; w	∼ 1,680; w	1,680–1,600 (w)
P=O aliphatic	1,152.52; m	1,150.59; m	1,150.59; m	1,150.59; m	∼ 1,150 (m)
Amide II	∼ 1,480; w	∼ 1,480; w	∼ 1,480; w	1,572.66; w	1,575–1,480 (w)

Abbreviations: br, broad; FT-IR, Fourier transform infrared; HA, hydroxyapatite; m, medium; PVA, polyvinyl alcohol; s, strong; sh, sharp; w, weak.

### Tensile Strength Test Results

The results mean ± standard deviation of the tensile strength test of the PVA-Collagen-HA composite membrane on dry condition without irradiation, 15 kGy irradiation, and 25 kGy irradiation, the each group was different, but the highest value was in the 25 kGy irradiation group. Analysis using the one-way ANOVA parametric test showed a significant difference ( *p* < 0.05) in each group of PVA–collagen–HA composite membranes ( *p* = 0.006). The analysis continued with the post hoc Tukey's test showed that there was no significant difference ( *p* > 0.05) between the PVA–collagen–HA composite membrane group without irradiation (control) with the 15 kGy irradiated groups ( *p* = 0.285) and the group without irradiation (control) with the 25 kGy irradiated groups ( *p* = 0.079). However, there was a significant difference ( *p* < 0.05) between the tensile strength values in the 15 kGy irradiated PVA–collagen–HA composite membrane group and the 25 kGy irradiated groups ( *p* = 0.005).

The analysis results of the mean ± standard deviation of the PVA-Collagen-HA composite membrane group on wet conditions in the group without irradiation, 15 kGy irradiation, and 25 kGy irradiation, the tensile strength of each group was different, with the highest value being the 25 kGy irradiation group. Data analysis continued by using the one-way ANOVA parametric test showed that there was no significant difference ( *p* > 0.05) in each group of PVA–collagen–HA composite membranes ( *p* = 0.373) ( Table 5 ).

**Table 5:** Results of mean ± SD and one-way ANOVA test on tensile strength test of PVA–collagen–HA composite membrane

Group	Total ( *n* )	SD tensile strength dry condition ± (MPa)	*p* -Value ANOVA dry condition	SD tensile strength wet condition ± (MPa)	*p* -Value ANOVA wet condition
PVA–collagen–HA composite membrane without radiation	5	32.86 ± 8.39 ^ab^	0.006	0.357 ± 0.111	0.373
15 kGy irradiated PVA–collagen–HA composite membrane *γ*	5	27.28 ± 4.38 ^b^		0.531 ± 0.285	
25 kGy irradiated PVA–collagen–HA composite membrane *γ*	5	41.28 ± 1.40 ^a^		0.537 ± 0.228	

Abbreviations: ANOVA, analysis of variance; HA, hydroxyapatite; PVA, polyvinyl alcohol, SD, standard deviation.

Note: Lowercase superscript letters “a and b” in different columns showed a significant difference ( *p* < 0.05).

### Porosity Test Results

**Fig. 3:**
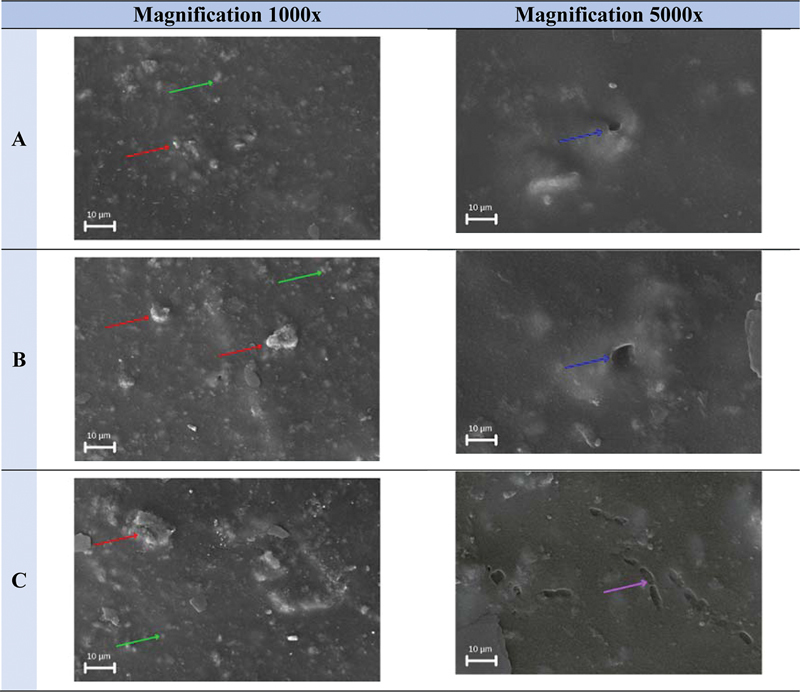
The results of the porosity test analysis on the polyvinyl alcohol–collagen– hydroxyapatite composite membrane group (A) without irradiation, (B) irradiation 15 kGy, and (C) irradiation 25 kGy with ×1,000 and ×5,000 magnification using scanning electron microscope. Red arrows indicate crystallization clumps. The green arrow shows the porous distribution. The blue arrows indicate the nonbonded porous structure. The purple arrow shows the interlocking porous structure.

The membrane surface in the three groups showed a smooth membrane surface, and there were crystallization lumps, both at ×1,000 and ×5,000 magnification. The porosity distribution was evenly distributed across the three groups of membranes, seen at ×1,000 magnification. The porous structures in the nonirradiated ( Fig. 3A ) and 15 kGy ( Fig. 3B ) irradiated groups were open but not bonded to each other, in the 25 kGy ( Fig. 3C ) irradiated groups; the porous structures were open and bonded to each other, seen at ×5,000 magnification.

The results of porosity measurements in each group were obtained using ImageJ software. In the three groups, the PVA–collagen–HA composite membrane showed a porous size with a microporous category having a porous size of ≤10 µm ( Table 6 ).

**Table 6:** Mean ± SD of porous size in each group of PVA–collagen–HA composite membrane using ImageJ software

Group	Mean ± SD porous size PVA–collagen–HA composite membrane
0 kGy	4.65 ± 2.70 µm
15 kGy	6.51 ± 5.78 µm
25 kGy	8.08 ± 7.32 µm

Abbreviations: HA, hydroxyapatite; PVA, polyvinyl alcohol, SD, standard deviation.

### Degradability Test Results

**Fig. 4:**
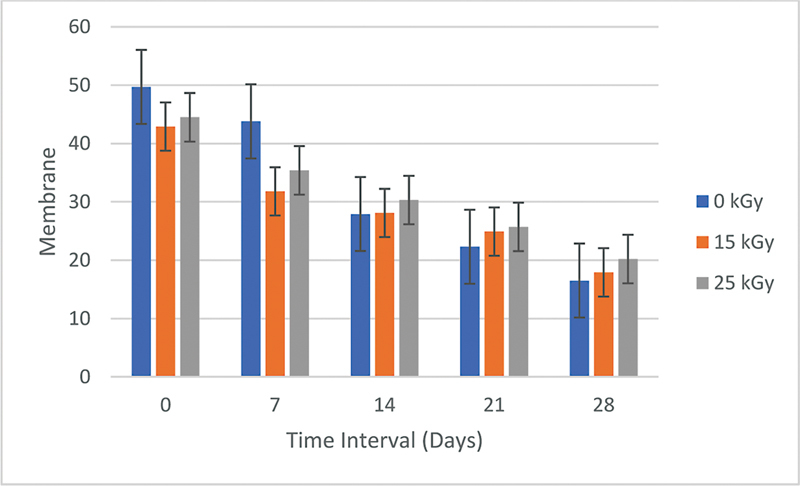
Graph of the results of the degradability test in each group of polyvinyl alcohol–collagen–hydroxyapatite composite membrane without irradiation, irradiation 15 kGy, and irradiation 25 kGy with time intervals of 0, 7, 14, 21, and 28 days.

**Fig. 5:**
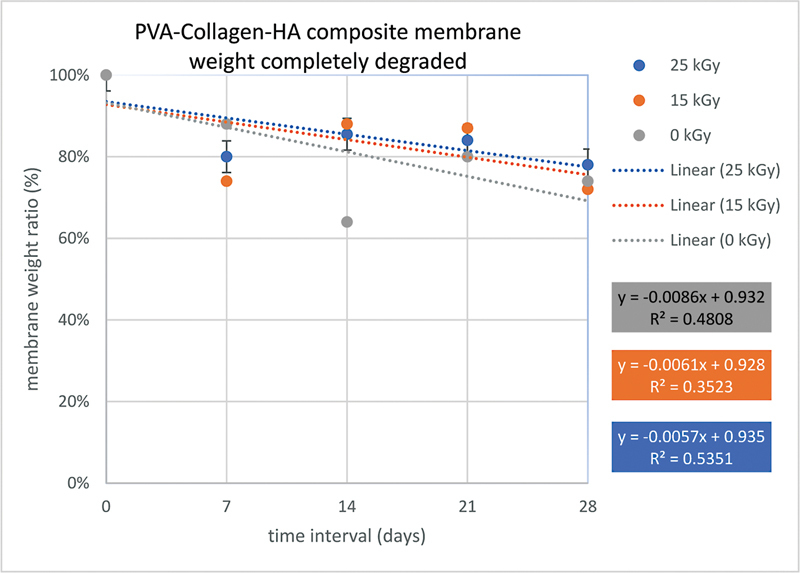
Linear graph of the PVA–collagen–HA composite membrane weight completely degraded. HA, hydroxyapatite; PVA, polyvinyl alcohol.

The results of the degradability test in each group of PVA–collagen–HA composite membranes without irradiation, 15 kGy, and 25 kGy immersed in PBS solution assessed at time intervals of 7, 14, 21, and 28 days showed a decrease in membrane weight as shown in Fig. 4 with the percentage weight of each group of PVA–collagen–HA composite membranes was calculated using the formula weight ratio (%) as shown in Table 7 . The results of the calculation of the total weight of the completely degraded PVA–collagen–HA composite membrane are shown in Fig. 5 .

**Table 7:** Weight ratio, mean ± SD, *p* -value one-way ANOVA, and post hoc Tukey's test of PVA–collagen–HA composite membrane group without irradiation, 15 kGy, and 25 kGy with time intervals of 7, 14, 21, and 28 days

Group		Repetition ( *n* )	Weight ratio (%)	Mean ± SD (%)	*p* -Value ANOVA
0 kGy	W7	6	88	43.83 ± 12.95 ^ac^	0.000
	W14	6	64	27.96 ± 4.12 ^abc^	
	W21	6	80	22.30 ± 3.39 ^abc^	
	W28	6	74	16.58 ± 2.19 ^abc^	
15 kGy	W7	6	74	31.80 ± 5.70 ^bc^	
	W14	6	88	28.13 ± 6.75 ^abc^	
	W21	6	87	24.93 ± 4.79 ^abc^	
	W28	6	72	17.96 ± 1.99 ^abc^	
25 kGy	W7	6	80	35.45 ± 3.17 ^abc^	
	W14	6	86	30.31 ± 4.54 ^abc^	
	W21	6	84	25.76 ± 3.97 ^abc^	
	W28	6	78	20.23 ± 5.54 ^abc^	

Abbreviations: ANOVA, analysis of variance; HA, hydroxyapatite; PVA, polyvinyl alcohol, SD, standard deviation.

Note: Lowercase superscript letters “a to c” in different columns showed a significant difference ( *p* < 0.05).

The mean ± standard deviation for each group of PVA–collagen–HA composite membranes is shown in Table 7 . One-way ANOVA test obtained a *p* -value = 0.000, so it could be concluded that there is a significant difference ( *p* < 0.05) in each treatment group PVA–collagen–HA composite membrane without irradiation, 15 kGy, and 25 kGy with an interval of 7, 14, 21, and 28 days. Tukey's post hoc test analysis showed that the weight of the PVA–collagen–HA composite membrane group without irradiation compared with the 15 kGy groups at 7-day intervals ( *p* = 0.023) showed that the results were significant ( *p* < 0.05). In contrast, the other group showed no significant difference ( *p* > 0.05) ( Table 7 ).

## Discussion

In this study, the PVA–collagen–HA composite membrane has a synthetic polymer, namely, PVA, which is biotribological with a surface that is resistant to friction and tension and has good chemical properties and degradability. 19 The PVA–collagen–HA composite membrane has natural polymers, namely, collagen and HA, with the advantages of being biocompatible and stimulating optimal wound healing. However, the lack of collagen and HA is that they have low mechanical properties and are easily degraded by sterilization. 20 Therefore, in this study, the synthetic polymer content of PVA is needed to strengthen the mechanical and physical properties of the PVA–collagen–HA composite membrane to support soft and hard tissues regeneration.

Chemical properties have been seen from the chemical structure in the FT-IR test phase 1 on the PVA–collagen–HA composite membrane group without irradiation, 15 kGy irradiation, and 25 kGy irradiation indicating the presence of identical absorption peaks of PVA, collagen, and HA functional groups that appeared. Each group of PVA–collagen–HA composite membranes showed a characteristic peak of PVA, namely, asymmetric CH and widening of the OH strain absorption peak due to intra- and extraintermolecular hydrogen bonds in the PVA–collagen–HA composite membrane. This is in line with previous research, which stated that the peak characteristic of PVA was found to be a stretching vibration of the -OH group, which experienced widening due to intra- and extrahydrogen bonds between molecules. 21 This bond shows a strong interaction between -OH from PVA and C=O from collagen to form an acetal bridge from CO, which reduces the content of free -OH groups. 22

The PVA–collagen–HA composite membrane group without irradiation (control), 15 kGy irradiation, and 25 kGy irradiation found the characteristic peaks of collagen in the bands of amide A, amide II, and amide III. Compared to the FT-IR graph, each group did not show a significant change in the characteristic peaks of collagen, proving that the collagen polymer's structure is not lost after gamma irradiation. In a previous study, the results of FT-IR collagen extraction from white snapper scales ( *L. calcarifer* ) were found in the amide A band, amide I band, and amide II band. 23 This proves that the collagen polymer content in the PVA–collagen–HA composite membrane is extracted from the scales of white snapper ( *L. calcarifer* ).

The PVA–collagen–HA composite membrane group without irradiation (control), 15 kGy irradiation, and 25 kGy irradiation showed the presence of aliphatic P=O groups. This is in line with previous studies, which showed that the characteristics of HA extraction from white snapper scales ( *L. calcarifer* ) showed the presence of aliphatic P=O groups. 24 In this study, it was proven that the HA polymer content in the PVA–collagen–HA composite membrane was extracted from the scales of white snapper ( *L. calcarifer* ). Compared with each group, there was no significant change in the characteristic peak of the HA polymer. This indicates that the structure of the HA polymer is not lost after gamma-ray irradiation.

The results of the second FT-IR test on the PVA–collagen–HA composite membrane without irradiation, 15 kGy irradiation, and 25 kGy irradiation, which were stored for 30 days on three media, showed the presence of functional groups similar to the functional groups that appeared on the FT-IR graph phase 1. This proves that the chemical structure of the PVA–collagen–HA composite membrane remains stable, and no functional groups are lost after storage on three different media for 30 days. The presence of synthetic polymer content PVA also supports the cross-linking reaction with free base hydroxyl groups, which can increase chemical stability in biomaterial storage. 25 This shows that the PVA polymer content strengthens the chemical structure stability of the PVA–collagen–HA composite membrane during 30 days of storage on three different media.

The tensile strength of the dry condition PVA–collagen–HA composite membrane showed that the 25 kGy irradiated groups had the highest value compared with the 15 kGy and no irradiated groups. The increase in tensile strength in the 25 kGy irradiation group indicated a PVA cross-linking reaction due to induction by gamma radiation. 23 Previous studies have shown that an increase in cross-linking reactions can reduce the crystallinity of polymer membranes so that the tensile strength value becomes higher. Meanwhile, the 15 kGy irradiated groups had lower tensile strength than those without radiation. This is contrary to previous studies, where there was an increase in tensile strength with an increase in the dose of gamma irradiation. 26 The decrease in tensile strength in the 15 kGy irradiation group indicated that the crystallinity of the membrane was increasing. This can occur because the bonds between compounds in gamma-ray irradiation cause an increase in molecular density. The lower tensile strength in the 15 kGy irradiated group was thought to be due to the unstable bond between PVA, collagen, and HA. This is in line with previous studies, which showed that homopolymerization could occur at specific doses of gamma irradiation, inhibiting cross-linking and decreasing tensile strength. 27 So, this study proves that the 25 kGy irradiated group has a more optimal tensile strength than the 15 kGy irradiation because it has stable bonds between compounds. The dry condition PVA–collagen–HA composite membrane has a high tensile strength ranging from 27.28 ± 8.39 to 41 ± 1.40 MPa. In the previous studies, the ideal GTR and GBR membranes in dry conditions had tensile strengths ranging from 22.5 to 40 MPa. 28 This proves that the dry condition PVA–collagen–HA composite membrane is in the optimal tensile strength range as GTR and GBR membranes.

Tensile strength PVA–collagen–HA composite membrane in wet conditions showed that the 25 kGy irradiated groups had the highest value, followed by the 15 kGy irradiated groups, and the lowest was the nonirradiated group. This is in line with previous studies, which showed that the tensile strength increased significantly with increasing the dose of gamma irradiation given. 26 The increase in tensile strength is due to increased cross-linking reactions, which can reduce the polymer membrane's crystallinity so that the tensile strength value becomes higher. 26 29 According to the International Standard ISO 11137, the optimum sterilization dose for medical biomaterials is 25 kGy. 30 This shows that the 25 kGy irradiated group has ideal characteristics as GTR and GBR membranes with the best tensile strength and the optimum sterilization dose. PVA–collagen–HA composite membrane in wet conditions had high tensile strength in the range of 0.357 ± 0.11 to 0.537 ± 0.22 MPa. A previous study showed that the ideal GTR and GBR membranes in wet conditions had tensile strengths ranging from 0.127 to 1.2 MPa. 31 This proves that the wet condition PVA–collagen–HA composite membrane is in the optimal tensile strength range as GTR and GBR membranes.

The results of the tensile strength test on the PVA–collagen–HA composite membrane showed that the dry condition was higher than the wet condition due to the effect of immersion in saline sterile NaCl solution. In the previous studies, the tensile strength of composite membranes in dry conditions was higher than in wet conditions. 7 Immersion in sterile saline NaCl solution can reduce the mechanical properties of the composite membrane because the matrix bonds are not tight, so they cannot withstand the tensile force given. In addition, immersion in saline sterile NaCl solution releases the bonding of the fiber surface (debonding) and the absorption of water molecules which causes the composite to swell (swelling). 32

In this study, the porous size in each group of the PVA–collagen–HA composite membrane showed a microporous size. The PVA–collagen–HA composite membrane, from the smallest to the largest, was the group without irradiation, 15 kGy irradiation, and 25 kGy irradiation. In the study, membranes with a microporous size were effective in regenerating bone compared with membranes with large pores. When the membrane is exposed to gamma-ray radiation, tiny pores are incorporated by cross-linking, which increases the intensity of the larger porous sizes. The combination of these tiny pores at a dose of 15 kGy resulted in a minimal increase in porous intensity; at a dose of 25 kGy, there was a maximum increase in porous intensity. 33 Based on research with membranes irradiated at a dose of 15 and 25 kGy irradiation, it was shown that membranes irradiated at a dose of 25 kGy had a larger porous size than those irradiated at a dose of 15 kGy. It was proven in this study that the larger the radiation dose, the larger the porous size. Although a dose of 25 kGy has a larger pore size than the group without irradiation and 15 kGy, it is still in the ideal category. In the previous studies, PVA–collagen–HA composite membranes at 15 and 25 kGy irradiation could be used as membranes with potential as GTR 15 and at 25 kGy radiation could be used as membranes with potential as GBR. 16

The PVA–collagen–HA composite membrane in this study could be degraded by decreasing the weight of the membrane every time interval of 7, 14, 21, and 28 days. The total weight of the completely degraded membrane after 28 days of immersion from the most degraded to the least, respectively, was the group without irradiation, 15 kGy irradiation, and 25 kGy irradiation. The group without irradiation experienced the most degradation due to the weak cross-links in the membrane so that the hydrogen elements were easily released, whereas in the 15 kGy irradiated and 25 kGy irradiated groups there were strong cross-links in the membrane, so that the hydrogen elements were not easily separated. 34 This also proves that all groups of membranes have ideal degradation of 4 to 6 weeks for soft tissue and 12 to 24 weeks for hard tissue. 12 13

The effect of gamma radiation on degradability test showed that the 25 kGy group had the longest degradation compared with the other groups. Gamma-ray radiation is not only used as a sterilizer for medical products but can also cross-link polymers. Cross-linking can improve mechanical properties and resistance to membrane degradation. In research using gamma-ray radiation, the higher the dose, the more cross-linking is achieved. 34 In a degradability test study with membranes irradiated at a dose of 15 and 25 kGy after immersion in PBS solution, the membranes irradiated with a dose of 25 kGy were degraded less than the dose of 15 kGy. 35 At an optimal dose of 25 kGy can increase the polymer's cross-linking, which provides structural strength and stability to the ion. When polymers are exposed to gamma radiation, structural changes occur accompanied by cross-linking between molecules. 26 The limitation of this study is needed to complete aspects of the mechanical properties of PVA–collagen–HA composite membrane such as compressive strength study.

## Conclusion

PVA–collagen–HA composite membranes showed a stable chemical structure during 30 days of storage on three different media, highest tensile strength values in the 25 kGy irradiation groups, ideal porous size, and degradability values in the 15 and 25 kGy groups. This composite membrane can be used as an alternative to the GBR/GTR membrane which is good at supporting the growth of soft tissue and bone tissue membrane.
